# Green metrics in chalcone synthesis via Claisen-Schmidt condensation: a critical mini review

**DOI:** 10.3389/fchem.2026.1909081

**Published:** 2026-09-01

**Authors:** Pamela Mendioroz, M. Julia Castro, Vanina Estrada, Darío C. Gerbino

**Affiliations:** 1 Planta Piloto de Ingeniería Química-PLAPIQUI (Universidad Nacional Del Sur-CONICET), Bahía Blanca, Argentina; 2 Instituto de Química Del Sur, (INQUISUR, CONICET-UNS), Departamento de Química, Universidad Nacional Del Sur, Bahía Blanca, Argentina; 3 Departamento de Ingeniería Química, Universidad Nacional Del Sur (UNS), Bahía Blanca, Argentina

**Keywords:** chalcones, Claisen-Schmidt condensation, green chemistry, green metrics, sustainable synthesis

## Abstract

Chalcones are important scaffolds in medicinal chemistry and functional materials, commonly synthesized via the Claisen–Schmidt condensation. In recent years, numerous methodologies have been proposed to improve the sustainability of this transformation, including solvent-free conditions, heterogeneous catalysis, alternative solvents, and process-intensification strategies. However, the lack of standardized and quantitative assessment often limits meaningful comparison between reported approaches. This mini review critically examines recent advances in sustainable Claisen–Schmidt condensations through the lens of green chemistry metrics, with particular emphasis on reaction mass efficiency, process mass intensity, E-factor, and specific energy consumption as complementary descriptors of process performance. By analyzing representative methodologies under comparable criteria, the review highlights inconsistencies in reporting practices and identifies key parameters that influence the overall environmental footprint of the process. Beyond summarizing current strategies, the article discusses methodological limitations, common sources of bias in metric evaluation, and ongoing debates regarding the actual sustainability of widely adopted approaches. Finally, perspectives are provided on the need for standardized reporting protocols, broader integration of energy-related metrics, and the adoption of data-driven and life cycle-oriented frameworks to enable more reliable benchmarking and the rational design of greener synthetic routes to bioactive chalcones.

## 1 Introduction

Chalcones (1,3-diaryl-2-propen-1-ones) represent a versatile class of α,β-unsaturated carbonyl compounds with a broad spectrum of biological activities, including antifungal, anthelmintic, anticancer, antiviral, antioxidant, and antidiabetic effects (Cheenpracha et al., 2006; Enoki et al., 2007; Godara et al., 2024; Turani et al., 2024). Their structural simplicity, combined with pharmacological potential, has positioned them as privileged scaffolds in medicinal chemistry and as key intermediates in the synthesis of flavonoids and other bioactive molecules (Alidmat et al., 2026). In addition, several chalcone derivatives have shown relevant enzyme inhibitory properties, including monoamine oxidase inhibition and other biologically relevant targets (Chimenti et al., 2010; Romagnoli et al., 2008; Sashidhara et al., 2010).

The Claisen–Schmidt condensation remains the most widely employed synthetic route to chalcones, typically involving base-catalyzed aldol condensation between acetophenone derivatives and aromatic aldehydes. Despite its operational simplicity and efficiency, conventional protocols often rely on homogeneous bases, organic solvents, and prolonged reaction times, leading to concerns regarding waste generation, process safety, and energy consumption. These limitations have driven the search for more sustainable alternatives in accordance with the principles of green chemistry.

In recent years, numerous strategies have been proposed to improve the sustainability of the Claisen–Schmidt condensation, including solvent-free methodologies, heterogeneous and recyclable catalysts, alternative reaction media such as ionic liquids, deep eutectic solvents, polyethylene glycol, and micellar systems, and process intensification techniques such as microwave and ultrasound irradiation (Adnan et al., 2020; Dotta et al., 2025; Lahyani et al., 2013; Qian et al., 2011; Shen et al., 2008; Tedesco et al., 2026; Yadav and Wagh, 2020). While many of these approaches are presented as greener alternatives, their environmental benefits are often inferred from isolated parameters such as reaction yield or reduced reaction time, rather than from comprehensive quantitative assessment. This has led to ongoing debate regarding the actual sustainability of widely adopted methodologies, particularly when parameters such as material efficiency and energy consumption are not systematically evaluated.

A major limitation in the current literature is the lack of standardized frameworks for assessing and reporting sustainability. In particular, inconsistent or incomplete reporting of mass efficiency, waste generation, and energy input hinders meaningful comparison across studies and may introduce bias in the interpretation of “green” performance. Green chemistry metrics—such as reaction mass efficiency (RME), process mass intensity (PMI), and E-factor—offer reliable tools to quantify material efficiency and environmental impact, yet their application in chalcone synthesis remains limited and often non-uniform. Moreover, energy consumption, especially in intensified processes, is rarely quantified despite its critical contribution to overall process sustainability (Adhi et al., 2024).

Therefore, there is a clear need for systematic and critical analyses capable of comparing different methodologies using quantitative sustainability criteria. To the best of our knowledge, no previous review has comprehensively evaluated representative Claisen–Schmidt condensation methodologies through the simultaneous application of mass-based and energy-based green metrics. To facilitate direct comparison between conventional and sustainable approaches, a representative homogeneous Claisen–Schmidt protocol was selected as a benchmark, and its green chemistry metrics were calculated using the same methodological assumptions applied to the sustainable methodologies discussed herein (Organic Syntheses, 1922; Yadav and Wagh, 2020). Accordingly, this Mini Review critically examines recent advances in sustainable chalcone synthesis, emphasizing the opportunities and limitations associated with the interpretation of green chemistry metrics. Ultimately, this study aims to contribute to the development of more robust benchmarking practices and to support the rational design of greener synthetic strategies for the preparation of biologically relevant molecules, in line with the fundamental principles of green chemistry (Anastas and Warner, 1998).

## 2 Sustainability metrics in organic synthesis

The development of sustainable synthetic methodologies requires robust and quantitative tools to evaluate their environmental impact. In this context, green chemistry metrics have emerged as valuable instruments for assessing material efficiency, waste generation, and overall process performance. Among these, reaction mass efficiency, process mass intensity, and the E-factor are the most widely applied indicators in organic synthesis (Constable et al., 2002; Sheldon, 2018).

RME is defined as the ratio between the mass of the desired product and the total mass of reactants, taking into account the stoichiometry of the reaction. This metric provides insight into how effectively the starting materials are incorporated into the final product (Sheldon, 2018; Trost, 1991). In contrast, PMI considers the total mass of all materials used in the process, including solvents, reagents, and auxiliary substances, relative to the mass of product obtained (Jiménez-González et al., 2011). As such, PMI offers a more comprehensive assessment of material usage at the process level. The E-factor, introduced by Sheldon, quantifies the amount of waste generated per unit mass of product and is particularly useful for benchmarking industrial and laboratory-scale processes (Sheldon, 2017).

Despite their widespread use, these metrics present inherent limitations. RME does not account for solvents or auxiliary substances, potentially overestimating the sustainability of processes that rely heavily on non-reactive materials. PMI, while more inclusive, may be sensitive to the definition of system boundaries, particularly regarding solvent recycling or work-up procedures. Similarly, the E-factor does not distinguish between different types of waste, thereby overlooking qualitative aspects such as toxicity or environmental persistence (Constable et al., 2002; Sheldon, 2018).

Importantly, the uncritical use of isolated metrics may lead to inconsistent or potentially misleading conclusions when comparing synthetic methodologies, particularly when different studies rely on non-equivalent system boundaries or incomplete datasets. Consequently, a comprehensive sustainability assessment should consider multiple complementary metrics rather than relying on a single indicator.

Although several more comprehensive sustainability assessment tools integrating environmental, safety, energy, and process-related criteria have also been proposed (Martínez et al., 2022; Van Aken et al., 2006), their application generally requires experimental information that is not consistently reported in the primary literature analyzed here. Moreover, several of these methodologies rely on qualitative or semi-quantitative assessments that cannot be reconstructed retrospectively from published experimental procedures. From a practical standpoint, the routine reporting of standardized mass-based metrics following established calculation protocols, such as those proposed in the *Green Chemistry Metrics Toolkit* (McElroy et al., 2015), would represent an important first step toward a more comprehensive sustainability assessment. Once consistent reporting practices become widespread, composite assessment frameworks could then be applied across different synthetic methodologies, using common methodological assumptions and system boundaries.

In addition to mass-based metrics, specific energy consumption (SEC) is especially relevant for evaluating electrically assisted process-intensification techniques, particularly microwave- or ultrasound-assisted reactions. Although energy efficiency is also an important consideration in continuous-flow and microreactor technologies, comparable energy consumption data are generally unavailable for the representative Claisen–Schmidt methodologies reported to date. Contrary to common assumptions, shorter reaction times do not necessarily translate into lower overall energy demand, and quantitative energy assessments remain relatively scarce in the literature (Adhi et al., 2024). Because reliable and sufficiently detailed energy consumption data are generally available only for electrically assisted methodologies, SEC comparisons in this review are restricted to microwave- and ultrasound-assisted systems and should not be directly extrapolated to conventionally heated or continuous-flow processes.

Another important challenge lies in the lack of standardized reporting practices. In many cases, incomplete experimental information—such as missing solvent quantities, unreported purification steps, undefined reaction scales, or insufficient details regarding catalyst recovery—limits the accurate calculation of green chemistry metrics and hinders meaningful cross-comparison between methodologies. These inconsistencies may introduce bias and contribute to misleading conclusions regarding the relative sustainability of different approaches.

Although this review focuses on the synthesis step for metric calculations, downstream work-up procedures varied considerably among the reported methodologies. In several cases, solvent-free or aqueous reactions were followed by solvent-intensive extraction or purification steps, whereas other protocols enabled simple filtration or direct crystallization. Since work-up operations may substantially affect the overall environmental footprint, more standardized reporting of isolation and purification procedures would facilitate more comprehensive sustainability assessments.

In the present review, representative Claisen–Schmidt condensation methodologies were evaluated using a common set of sustainability metrics whenever sufficient experimental information was available. Calculations were performed from data reported in the original publications, and any assumptions regarding catalyst reuse, solvent inclusion, or reaction scale are explicitly indicated when applicable. This harmonized approach seeks to improve comparability among studies and to facilitate a more objective interpretation of reported sustainability claims.

Therefore, while green chemistry metrics provide a valuable framework for evaluating synthetic methodologies, their meaningful application requires careful consideration of system boundaries, transparent reporting, and critical interpretation of results. In the context of chalcone synthesis via Claisen–Schmidt condensation, the systematic application of these metrics enables a more rigorous assessment of sustainability claims and facilitates the identification of genuinely improved synthetic strategies.

## 3 Green strategies for Claisen–Schmidt condensation

Numerous approaches have been developed to improve the sustainability of the Claisen–Schmidt condensation, including solvent-free protocols, heterogeneous catalysis, alternative reaction media, and process-intensification strategies. While many of these methodologies are frequently described as “green”, direct comparison is often complicated by differences in reaction conditions, catalyst loading, substrate scope, system boundaries, and reporting practices. To facilitate a more objective assessment, representative examples from the literature were analyzed using the sustainability metrics discussed above, with particular emphasis on material efficiency and, when available, energy consumption. Only methodologies providing sufficient experimental information to enable recalculation of the harmonized green chemistry metrics under the common benchmark framework adopted in this review were selected for detailed comparative analysis. For studies reporting multiple catalysts, catalyst formulations, or optimized reaction conditions, a single representative system was selected. The discussion is organized according to the main catalytic systems and process-intensification strategies reported in the literature, allowing a more systematic comparison of their sustainability performance.

**FIGURE 1:**
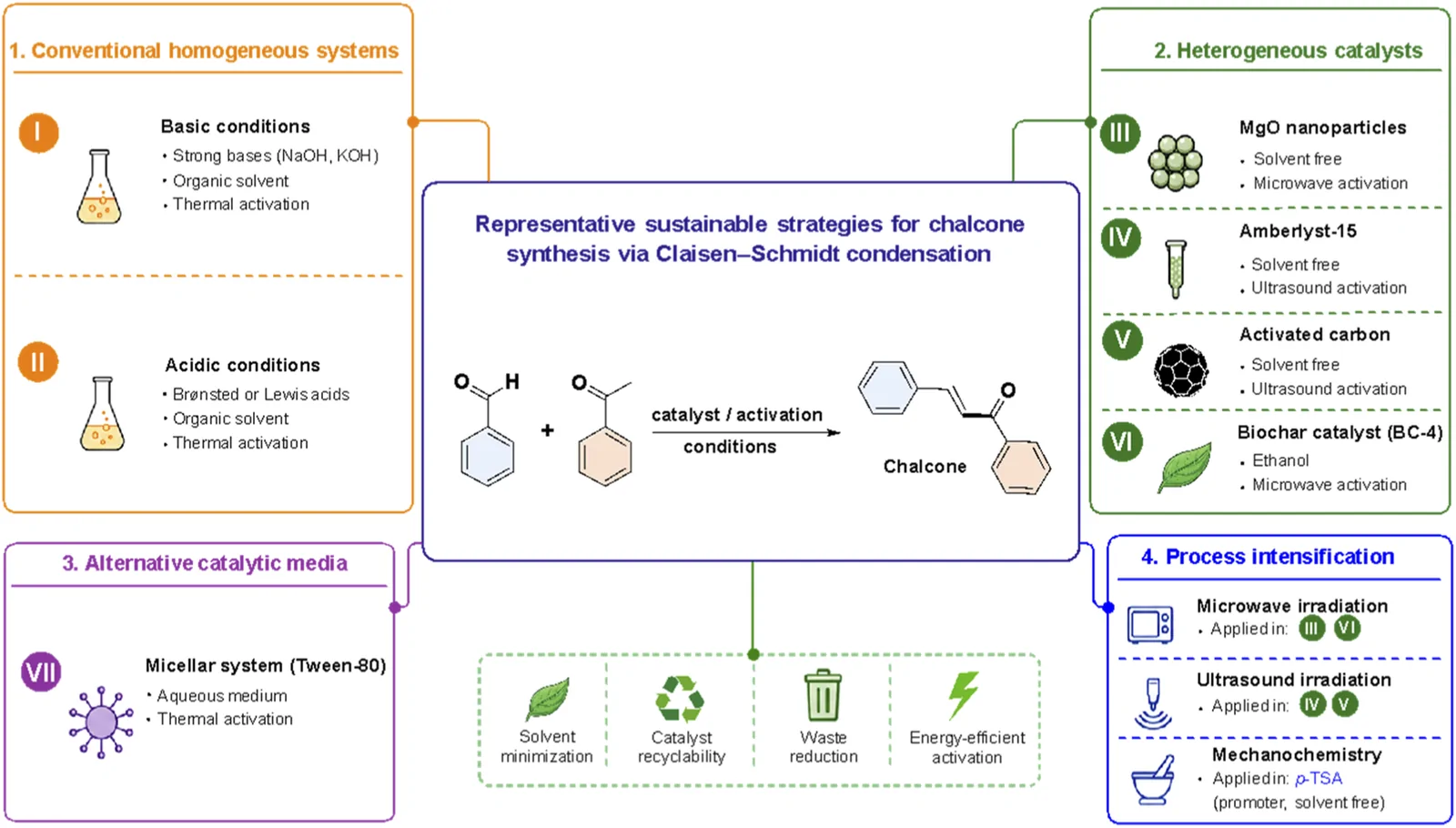
Overview of the representative conventional and sustainable strategies for chalcone synthesis via Claisen–Schmidt condensation analyzed in this review. The methodologies are organized according to catalyst class, reaction medium, and process-intensification strategy, highlighting the representative systems selected for comparative evaluation using harmonized green chemistry metrics. Roman numerals (**I**–**VI**) identify the representative methodologies discussed throughout Section 3 and summarized in Table 1.

Representative reaction conditions and the corresponding green chemistry metrics are summarized in Table 1, whereas the main green strategies discussed throughout this section are illustrated in Figure 1.

**TABLE 1:** Comparison of representative conventional and sustainable Claisen–Schmidt condensation methodologies using a common benzaldehyde–acetophenone benchmark reaction, including representative reaction conditions and calculated green chemistry metrics.

Catalytic system	Reaction medium	Representative reaction conditions	Yield (%)	RME (%)	PMI	E-factor	SEC (kWh kg^-1^)	Catalyst reuse (cycles)	Ref.
Homogeneous KOH (benchmark)	Ethanol	Conventional heating, 15 °C–30 °C, 12 h	97.0	89.3	4.54	3.54	–^c^	–	Organic Syntheses (1922)
MgO nanopowder	Solvent-free	Microwave irradiation, 150 °C, 120 min	59.0	54.3	1.89	0.89	6.5 × 10^3^	5	Tedesco et al. (2026)
Amberlyst-15	Solvent-free	Ultrasound irradiation, 60 °C, 60 min	98.0	76.0	1.61	0.61	5.5 × 10^4^	4	Lahyani et al. (2013)
Tween-80 micellar system	Micellar medium (H_2_O)	Conventional heating, 45 °C, 24 h	84.0	77.3	1.41	0.41	1.6 × 10^4^	3^a^	Dotta et al. (2025)
Biochar (BC-4)	Ethanol	Microwave irradiation, 60 °C, 25 min	93.0^b^	79.9	10.20	9.20	4.3 × 10^2^	5	Turani et al. (2024)
Activated carbon	Solvent-free	Ultrasound irradiation, 30 °C, 120 min	75.0	69.0	1.83	0.83	1.4 × 10^3^	n.r.	Calvino et al. (2006)
*p*-TSA	Solvent-free	Mechanochemical grinding, rt, 5 min	97.0	89.3	2.06	1.06	–^c^	–	Adnan et al. (2020)

Green chemistry metrics (AE, RME, PMI, E-factor and SEC) were recalculated by the authors following the methodology proposed in the Green Chemistry Metrics Toolkit (McElroy et al., 2015) using harmonized calculation criteria. The conventional homogeneous KOH protocol included in this table corresponds to the benchmark reaction described in Organic Syntheses (1922). Complete reaction conditions, reaction scales, reagent quantities, catalyst loadings, assumptions, and calculation worksheets are provided in Supplementary Information S1 and Supplementary File S2.

^a^ Catalyst reuse experimentally demonstrated for three consecutive reaction cycles using a different substrate; similar behavior was assumed for the model reaction.

^b^ Highest isolated yield reported for the benchmark reaction between benzaldehyde and acetophenone.

^c^ SEC could not be calculated because the original publication did not provide sufficient information to estimate the energy consumption.

### 3.1 Conventional methodology

The classical synthesis of chalcones is predominantly achieved through the Claisen–Schmidt condensation between an aromatic aldehyde and an acetophenone derivative, corresponding to a crossed aldol condensation followed by dehydration. This transformation is typically carried out under homogeneous basic or acidic conditions, frequently employing strong bases in alcoholic solvents such as ethanol or methanol (Mulugeta, 2022). Acid-catalyzed variants have also been reported using Brønsted acids such as HCl and *p*-toluenesulfonic acid, as well as Lewis acids including AlCl_3_, TiCl_4_, and BF_3_ (Lahyani et al., 2013; Narender and Reddy, 2007).

As a representative benchmark for comparison, the classical Claisen–Schmidt condensation between benzaldehyde and acetophenone under homogeneous basic conditions was selected. The benchmark protocol was based on the widely adopted KOH/ethanol procedure described in Organic Syntheses (1922), which remains one of the most representative laboratory methods for chalcone synthesis and has been recognized as a reference conventional methodology in recent critical reviews of green Claisen–Schmidt condensations (Organic Syntheses, 1922; Yadav and Wagh, 2020). To ensure a meaningful comparison, the corresponding green chemistry metrics were calculated for the benchmark reaction between benzaldehyde and acetophenone using the same methodological assumptions applied to all sustainable methodologies discussed throughout this review.

For consistency with the harmonized system boundaries adopted throughout this review, the benchmark calculations were based on the reported crude yield described in the original *Organic Syntheses* procedure, whereas the subsequent recrystallization step was considered part of the downstream work-up and was therefore excluded from the assessment, in accordance with the methodological criteria applied to all representative methodologies evaluated. Despite its operational simplicity and generally good yields, this conventional benchmark methodology presents several limitations from both synthetic and environmental perspectives. Reaction times may be prolonged, selectivity can be affected under strongly basic conditions, and the reliance on homogeneous bases, organic solvents, and stoichiometric work-up operations contributes significantly to material consumption and waste generation. These characteristics are reflected in the comparatively high PMI and E-factor values calculated for the benchmark reaction (Table 1), providing a quantitative baseline against which the sustainable methodologies discussed throughout this review can be objectively evaluated. These limitations have stimulated the development of alternative catalytic systems, alternative reaction media, and process-intensification strategies aimed at improving material efficiency, minimizing waste generation, and reducing energy consumption. The following sections critically examine these approaches using a common set of green chemistry metrics, with the conventional homogeneous protocol serving as the reference benchmark for comparison.

### 3.2 Catalytic systems

Efforts to overcome the limitations of conventional homogeneous Claisen–Schmidt condensations have focused primarily on the development of heterogeneous catalytic systems that improve catalyst recovery, facilitate product isolation, and reduce solvent consumption. These approaches range from conventional solid acid/base catalysts to nanostructured materials and alternative catalytic media, frequently combined with process-intensification strategies such as microwave or ultrasound irradiation.

#### 3.2.1 Solid acid/base catalysts

Conventional heterogeneous solid acid and base catalysts constitute one of the most extensively investigated alternatives to homogeneous catalysis because they facilitate catalyst recovery while reducing corrosion and simplifying product purification. Numerous heterogeneous catalysts have been reported for Claisen–Schmidt condensations, including layered double hydroxides (hydrotalcites), zeolites, hydroxyapatites, ion-exchange resins, and supported metal oxides (Climent et al., 1995; Yadav and Wagh, 2020). Among these, the Amberlyst-15 methodology reported by Lahyani et al. (2013) was selected as a representative example because it provides sufficient experimental information to allow recalculation of the green chemistry metrics using the harmonized methodology adopted throughout this review.

Lahyani et al. (2013) described the use of ion-exchange resins such as Amberlyst-15 under solvent-free conditions combined with ultrasound irradiation as part of intensified heterogeneous catalytic systems.

The methodology afforded chalcones in good to excellent yields (67%–98%) at moderate temperatures (60 °C) within 1 h, and the catalyst could be reused up to four cycles. For the benchmark reaction, the very good average yield (75%) over cycles corresponds to an RME of 76.0%. When only the synthetic step is considered, the methodology displays favorable material efficiency due to its solvent-free conditions and catalyst recyclability. Although a relatively high catalyst loading (0.4 g) was employed, the catalyst was reused 4 times throughout the recycling study, resulting in a PMI of 1.61 and an E-factor of 0.61. It should be noted, however, that the authors did not report the catalyst recovery by mass after each cycle, preventing a more rigorous assessment of catalyst losses during reuse. Furthermore, the calculated SEC is strongly influenced by the very small reaction scale employed and by the use of nominal ultrasound power values, emphasizing the limitations of direct energy-based comparisons under laboratory-scale conditions.

#### 3.2.2 Nanocatalysts

Nanostructured catalysts have attracted considerable attention due to their high surface area and tunable catalytic properties. A broad range of nanostructured catalysts has been investigated for Claisen–Schmidt condensations, including MgO, ZnO, TiO_2_, hydroxyapatite-based nanomaterials, magnetic Fe_3_O_4_-supported catalysts, and hierarchical porous MgO materials (Yadav and Wagh, 2020; Yang et al., 2016). The MgO nanopowder methodology reported by Tedesco et al. (2026) was selected as a representative example because complete experimental information was available for the harmonized comparative analysis performed in this review.

Tedesco et al. (2026) reported the use of MgO nanopowder as a recyclable heterogeneous catalyst for solvent-free Claisen–Schmidt condensation under microwave irradiation. The system accommodates both aromatic and biomass-derived aldehydes, including furanic substrates, highlighting its versatility.

Under microwave conditions (150 °C, 120 min), chalcone derivatives were obtained with high selectivity and yields up to 85%. The catalyst was reusable for up to five cycles with minimal activity loss. For the benchmark reaction involving benzaldehyde, a 59% isolated yield was considered, corresponding to an RME of 54.3% when catalyst reuse is accounted for. The adjusted PMI and E-factor were estimated at 1.89 and 0.89, respectively.

Although the solvent-free conditions and catalyst recyclability contribute positively to material efficiency, the relatively moderate yield limits overall performance. Additionally, the absence of reported microwave power required estimation of the SEC, which was approximated based on typical reactor operation. These results illustrate both the advantages and the current limitations associated with nanostructured heterogeneous catalysts for sustainable Claisen–Schmidt condensations.

#### 3.2.3 Alternative catalytic media

Alternative reaction media such as micellar systems can act simultaneously as solvents and reaction promoters. Whereas micellar catalysis relies on substrate compartmentalization within surfactant aggregates, ionic liquids (ILs) and deep eutectic solvents (DESs) frequently act simultaneously as reaction medium and promoter through their intrinsic physicochemical properties.

Besides aqueous micellar systems, several environmentally benign reaction media have been explored for Claisen–Schmidt condensations, including ionic liquids, deep eutectic solvents, polyethylene glycol, surfactant-based systems, and other aqueous reaction media (Shen et al., 2008; Smith et al., 2014; Welton, 1999; Yadav and Wagh, 2020). Representative examples of these alternative media include acidic DESs capable of promoting the condensation without additional catalysts (Tiecco et al., 2016) and sulfonic acid-functionalized ionic liquids that simultaneously act as solvent and Brønsted acid catalyst while allowing medium recycling (Qian et al., 2011).

Although these alternative media have emerged as promising strategies for sustainable organic synthesis, the available literature generally does not provide sufficient experimental information to enable recalculation of harmonized green chemistry metrics using the standardized methodology adopted in this review. Information regarding solvent quantities, recovery efficiencies, downstream purification operations, or auxiliary materials is frequently incomplete, preventing direct comparison under the harmonized benchmark framework adopted throughout this review.

Among the methodologies identified in the literature, only the Tween-80 micellar system reported by Dotta et al. (2025) fulfilled these requirements. It was therefore selected as the representative case study because it provides sufficiently complete experimental information to enable transparent recalculation of all green chemistry metrics within the same harmonized benchmark framework.

The reported reactions proceeded in water at moderate temperatures (45 °C), affording products in good to excellent yields (78%–95%), although relatively long reaction times (24 h) were required. For the benchmark system, the reported 84% yield corresponds to an RME of 77.3%, with calculated PMI and E-factor values of 1.41 and 0.41, respectively, when considering only the synthetic step.

A notable aspect of this study is the explicit comparison between conventional and micellar systems, illustrating how differences in reaction media and the definition of system boundaries can significantly influence the calculated green chemistry metrics.

### 3.3 Process intensification strategies

In addition to catalyst design and reaction medium optimization, process intensification has emerged as an important strategy for improving the sustainability of Claisen–Schmidt condensations. Microwave irradiation, ultrasound, and mechanochemical activation are among the most extensively investigated approaches because they can reduce reaction times, improve energy efficiency, and minimize solvent consumption. More recently, continuous-flow processing and microreactor technology have also attracted considerable attention in sustainable organic synthesis owing to their enhanced heat and mass transfer, improved process safety, and scalability (Hessel et al., 2005; Plutschack et al., 2017). Furthermore, Pucihar et al. (2025) recently reported an automated microflow platform coupled with machine-learning algorithms capable of autonomously optimizing reaction temperature, residence time, and reactant flow rates to maximize product formation.

This work exemplifies a recurring reporting pattern in studies focused on process intensification, where the emphasis is placed on iterative process optimization rather than on the description of a single benchmark synthetic protocol. Experimental data are typically reported as concentration profiles, conversions, and process variables instead of the complete mass-based inventories required for harmonized sustainability calculations. As a result, it was not possible to recalculate the green chemistry metrics using the standardized methodology adopted throughout this review.

This also reflects a broader distinction between studies primarily focused on synthetic methodology and those centered on process engineering. While the latter generally emphasize reactor performance, process control, and process optimization, they do not always report the experimental information required for standardized green metrics assessment.

Consequently, despite their considerable potential for sustainable process development, only methodologies providing the minimum experimental dataset required for consistent green chemistry metrics calculations were considered for the comparative analysis presented in Table 1.

#### 3.3.1 Microwave-assisted reactions

Microwave irradiation enables rapid and homogeneous heating, often leading to shorter reaction times and improved efficiency. Turani et al. (2024) reported a microwave-assisted synthesis using biochar-based catalysts derived from biomass, highlighting the integration of renewable catalytic materials with microwave activation. This combination illustrates how catalyst design and process intensification can act synergistically to improve process sustainability.

Under relatively mild conditions (200 W, 60 °C, 25 min), chalcones were obtained in high yields (77%–95%), and the catalyst was reusable up to four cycles. For the benchmark reaction, the 93% yield corresponds to an RME of 79.9%, with PMI and E-factor values of 10.20 and 9.20, respectively, reflecting the contribution of ethanol as solvent.

In contrast to other systems, the SEC was comparatively low due to the short reaction time and efficient energy transfer, illustrating the advantages of microwave-assisted processes in terms of energy efficiency.

#### 3.3.2 Ultrasound-assisted reactions

Ultrasound irradiation promotes acoustic cavitation, generating localized high temperatures and pressures that enhance mass transfer. Calvino et al. (2006) investigated Claisen–Schmidt condensation using alkaline-doped activated carbons under ultrasound irradiation.

The reactions proceeded under mild conditions (30 °C–50 °C) in solvent-free systems, affording chalcones with conversions of up to 75%. The corresponding RME was 69.0%, with PMI and E-factor values of 1.83 and 0.83, respectively.

While material efficiency is favorable due to solvent-free conditions, the SEC remains relatively high compared to microwave-assisted systems, reflecting longer irradiation times and equipment characteristics. Consequently, the environmental advantages associated with ultrasound-assisted synthesis should be interpreted together with the corresponding energy requirements.

#### 3.3.3 Solvent-free mechanochemical approaches

Solvent-free methodologies represent one of the most direct strategies to reduce environmental impact. Adnan et al. (2020) reported a rapid mechanochemical synthesis using mechanochemical activation under solvent-free conditions, using *p*-TSA as promoter.

The reaction proceeds within minutes under manual grinding conditions, affording high yields (97%). The corresponding RME is 89.3%, with PMI and E-factor values of 2.06 and 1.06, respectively.

Although mechanochemical activation provides one of the highest material efficiencies among the methodologies evaluated, the stoichiometric use of *p*-TSA prevents this protocol from being considered a fully catalytic process. Additionally, the absence of experimentally reported energy consumption precludes direct comparison of SEC with electrically assisted methodologies.

## 4 Comparative analysis of green metrics and process performance

To enable a quantitative comparison among the representative methodologies discussed above, the selected case studies were evaluated using a common benzaldehyde–acetophenone benchmark reaction and a consistent calculation protocol. The resulting metrics are summarized in Table 1. The inclusion of a representative conventional homogeneous protocol (Organic Syntheses, 1922) provides a reference point against which the advantages and limitations of the alternative systems can be assessed. The discussion below focuses on the influence of the catalytic system, reaction medium, and process conditions on the calculated sustainability metrics.

As expected for Claisen–Schmidt condensations, atom economy is intrinsically high due to the stoichiometry of the condensation reaction itself (Trost, 1991; Yadav and Wagh, 2020). Consequently, the observed differences arise primarily from solvent use, catalyst loading, auxiliary reagents, and catalyst reuse, rather than from intrinsic reaction efficiency.

Reaction yield alone proved to be a poor predictor of sustainability. Although the conventional homogeneous KOH protocol reported in Organic Syntheses (1922) afforded one of the highest yields (97%) and, consequently, one of the highest RMEs (89.3%), its material efficiency was penalized by the large quantities of ethanol employed as reaction medium. Interestingly, the mechanochemical protocol reported by Adnan et al. (2020) achieved an identical RME under solvent-free conditions, whereas others like MgO-based methodology by Tedesco et al. (2026) exhibited a considerably lower RME (54.3%) because the benchmark reaction provided only moderate yield despite otherwise favorable process characteristics.

It should be emphasized that a single representative catalytic system was selected from each publication and all metrics were calculated using the common benzaldehyde-acetophenone benchmark reaction whenever possible.

For example, the methodology developed by Tedesco et al. (2026) was specifically designed to extend solvent-free microwave-assisted Claisen–Schmidt condensations to biomass-derived furanic aldehydes, while the catalyst reported by Turani et al. (2024) was prepared from biochar obtained by biomass waste pyrolysis. Likewise, Lahyani et al. (2013) also evaluated Amberlyst-200C alongside Amberlyst-15, obtaining comparable catalytic performance over repeated reuse cycles, whereas Amberlyst-15 was selected as the representative catalyst because it served as the reference system for the quantitative comparison adopted throughout this review. Consequently, the comparative metrics do not necessarily capture all sustainability advances reported in the original works.

Unlike RME, which is largely governed by product yield, PMI and E-factor explicitly account for all material inputs and therefore provide greater discrimination among the evaluated methodologies. The micellar methodology reported by Dotta et al. (2025) afforded the lowest PMI (1.41) and E-factor (0.41) followed by the Amberlyst-15 system reported by Lahyani et al. (2013) (1.61; 0.61), the activated carbon catalyst reported by Calvino et al. (2006), and the MgO nanopowder of Tedesco et al. (2026) (1.89; 0.89), demonstrating the benefits of solvent-free conditions and alternative reaction media, particularly when combined with catalyst reuse.

In contrast, the microwave-assisted biochar system described by Turani et al. (2024) exhibited comparatively high PMI (10.20) and E-factor (9.20) despite its excellent yield (93%) and associated RME (79.9%). Examination of the material inventory (see Supplementary File S2) shows that this behavior is largely attributable to the relatively large solvent-to-substrate ratio of ethanol used as reaction medium: if ethanol is excluded from the inventory, the calculated PMI for reactants, reagents, and catalyst according to McElroy et al. (2015) decreases to 1.47, placing this methodology among the best-performing systems evaluated. Under these considerations, the methodology is only surpassed by the conventional benchmark (1.37, excluding reaction media) and the micellar system reported by Dotta et al. (2025) (1.41, even considering all reported synthesis inputs).

These observations highlight solvent choice as a dominant factor governing resource efficiency. Solvent-free methodologies consistently afforded the lowest PMI and E-factor values, whereas use of relatively large solvent volumes increases both, even when the solvent itself was considered relatively benign. This reflects an inherent limitation of mass-based metrics, which assign equal weight to all material inputs regardless of their intrinsic hazard profile. PMI and E-factor quantify resource consumption rather than chemical hazard and complementary solvent selection frameworks, such as the solvent guide proposed by Henderson et al. (2011), provide valuable additional context.

Catalyst reuse represents another key parameter influencing the sustainability of heterogeneous catalytic systems; however, its evaluation remains inconsistent across the reviewed methodologies. Amberlyst-15 (Lahyani et al., 2013) and the BC-4 biochar catalyst (Turani et al., 2024) were assessed through successive reuse cycles by monitoring product yield, whereas MgO nanopowder (Tedesco et al., 2026) was evaluated primarily in terms of catalyst recovery efficiency. Likewise, Dotta et al. (2025) investigated the reuse of the micellar reaction medium without quantifying surfactant recovery. Consequently, catalyst activity and catalyst recovery are rarely reported simultaneously, limiting direct comparison among catalytic systems and introducing uncertainty into PMI and E-factor calculations.

Energy consumption introduces an additional and often underexplored dimension of sustainability. The calculated specific energy consumption spans several orders of magnitude (4.3 × 10^2^ to 5.5 × 10^4^ kWh kg^-1^), largely reflecting the small reaction scales typically employed in methodological studies. At millimolar scale, the nominal power of laboratory equipment becomes disproportionately large relative to the mass of product obtained, leading to inflated SEC values. Consequently, these values should be interpreted as comparative indicators rather than absolute measures of process efficiency. SEC comparisons are therefore restricted to electrically assisted methodologies.

Likewise, PMI and E-factor values remain dependent on the selected system boundaries, particularly regarding catalyst reuse, solvent recovery, and work-up operations. Differences in these assumptions may significantly affect the calculated values and should therefore be carefully considered when comparing methodologies from different studies.

Within the limitations discussed above, microwave-assisted systems such as that reported by Turani et al. (2024) exhibit comparatively low SEC values due to the combination of moderate power input and short reaction times. In contrast, prolonged ultrasound irradiation, particularly at very small scale as in Lahyani et al. (2013), results in significantly higher estimated energy demand. These observations further demonstrate that the sustainability of a Claisen–Schmidt methodology cannot be assessed using a single performance metric. Rather, catalyst type, reaction medium, catalyst reusability, material efficiency, and energy demand should be considered collectively to provide a balanced evaluation of the overall environmental performance.

Furthermore, the comparative analysis indicates that no single methodology simultaneously maximizes all green chemistry metrics. Rather, each approach combines distinct advantages and limitations depending on the sustainability criteria considered.

The micellar methodology reported by Dotta et al. (2025) provides the lowest PMI and E-factor, indicating the highest overall material efficiency. The solvent-free Amberlyst-15 system (Lahyani et al., 2013) combines excellent yield with catalyst reusability and similarly favorable material efficiency, although at relatively high catalyst loading. The MgO nanopowder methodology developed by Tedesco et al. (2026) achieves competitive PMI and E-factor values while additionally offering solvent-free operation, catalyst recyclability, and applicability to biomass-derived substrates beyond the benchmark reaction. The activated carbon catalyst reported by Calvino et al. (2006) also exhibits an attractive balance between material efficiency and operational simplicity. Meanwhile, the mechanochemical protocol described by Adnan et al. (2020) reaches the highest RME under solvent-free conditions, although its material efficiency is partly limited by the stoichiometric use of *p*-TSA. In contrast, the biochar-supported catalyst reported by Turani et al. (2024) presents comparatively high PMI and E-factor values owing to solvent consumption, despite combining excellent yield, the lowest SEC among the electrically assisted methodologies, catalyst recyclability, and the use of a catalyst derived from biomass waste.

Overall, the comparison indicates that solvent-free catalytic systems tend to provide the most favorable process efficiency, whereas energy performance is strongly dependent on experimental scale. Importantly, the data summarized in Table 1 demonstrate that improvements in individual performance indicators (e.g., yield or RME) do not necessarily translate into overall sustainability gains. These observations reinforce the need for integrated, multi-parameter evaluation frameworks supported by transparent reporting practices and clearly defined system boundaries for the meaningful assessment of green synthetic methodologies. Accordingly, meaningful comparison among sustainable Claisen–Schmidt methodologies requires standardized reporting practices and harmonized evaluation criteria applicable across different catalytic systems.

This distinction also illustrates an important conceptual difference between reaction-oriented and process-oriented sustainability metrics. Whereas RME primarily reflects the intrinsic material efficiency of the chemical transformation, PMI and E-factor additionally account for solvents, catalysts, and other auxiliary materials that substantially influence the overall environmental footprint. Consequently, a reaction exhibiting excellent stoichiometric performance does not necessarily correspond to an efficient chemical process. Rather, meaningful sustainability assessment requires the complementary interpretation of reaction- and process-level metrics within a harmonized evaluation framework.

## 5 Perspectives and future directions

Despite the considerable progress achieved in the development of greener methodologies for chalcone synthesis, one of the most evident limitations remains the inconsistent reporting of sustainability-oriented indicators. Key parameters such as catalyst loading and recovery, solvent quantities, and reaction scale are often described in non-standardized ways, which complicates data interpretation and limits meaningful comparison across studies, even at the level of the synthetic step.

A broader limitation arises from the intrinsic nature of commonly used green chemistry metrics. This distinction is particularly evident when comparing reaction-based metrics, such as RME, with process-based indicators including PMI and E-factor. A methodology exhibiting excellent reaction efficiency does not necessarily correspond to a more sustainable overall process, reinforcing the importance of interpreting complementary green chemistry metrics collectively rather than individually. Furthermore, these process-based metrics assign equal weight to all material inputs, regardless of their environmental or toxicological profiles. From a sustainability standpoint, however, significant differences exist between solvent classes. For instance, the use of relatively benign solvents such as ethanol is not equivalent to that of more hazardous alternatives such as chlorinated solvents. The incorporation of weighting factors or standardized solvent selection frameworks could therefore enhance the interpretative value of these metrics and provide a more realistic assessment of environmental impact.

An additional opportunity for improving comparability lies in the adoption of benchmark reactions. The use of common model substrates, standardized reaction scales, and reporting protocols would facilitate objective comparison among methodologies and reduce uncertainties associated with substrate-dependent effects. Beyond the use of common benchmark reactions, meaningful standardization also requires consistent calculation criteria and defined system boundaries. Differences in catalyst allocation, treatment of solvent recovery, inclusion or exclusion of downstream purification operations, and assumptions regarding auxiliary materials may substantially influence the calculated green chemistry metrics, even when identical synthetic transformations are evaluated. Accordingly, transparent methodological reporting is essential to distinguish genuine improvements in sustainability from differences arising solely from inconsistent assessment procedures.

The increasing adoption of process intensification strategies, including continuous-flow processing, microreactor technology, and automated optimization platforms, also introduces new challenges. While such approaches offer attractive opportunities to improve heat and mass transfer, process safety, scalability, and resource efficiency, their reporting is often oriented toward reactor performance and optimization variables rather than the mass-based inventories required for green chemistry metrics calculations. Future research and reporting guidelines would benefit from bridging the traditional divide between synthetic methodology and process engineering, incorporating experimental datasets that enable process optimization and quantitative sustainability assessment.

Energy consumption represents an additional dimension that remains insufficiently explored. As observed through the SEC analysis, values derived from millimolar-scale laboratory reactions may vary widely due to the disproportionate influence of equipment power relative to the small quantities of product obtained. Although such values should be interpreted with caution, they still provide useful comparative insights into different reaction platforms. Future studies should prioritize reporting energy input under clearly defined conditions and, whenever possible, evaluate processes at larger and more representative scales.

Another essential aspect concerns the definition of system boundaries. The inclusion or exclusion of solvents, work-up procedures, and auxiliary reagents can significantly affect calculated metrics, as illustrated by the discrepancies observed in micellar and conventional systems. Establishing standardized criteria for metric calculation would therefore improve reproducibility and facilitate cross-study benchmarking.

Looking forward, the integration of green chemistry metrics into routine methodological reporting should be encouraged as a standard practice rather than an optional complement. Moreover, combining mass-based metrics with energy analysis and, ultimately, life cycle assessment approaches could provide a more comprehensive evaluation of process sustainability. Such analyses would enable consideration of upstream and downstream impacts associated with catalyst production, solvent manufacture, energy generation, and waste treatment, aspects that remain invisible when only reaction-level metrics are considered.

The conclusions drawn from the present comparative analysis are consistent with broader observations reported in recent reviews on the application of green chemistry metrics and sustainability assessment in synthetic organic synthesis (Kernaghan et al., 2024; Martínez et al., 2022). These studies likewise emphasize that meaningful sustainability assessment requires the complementary interpretation of multiple metrics together with transparent system boundaries and standardized reporting practices. Consequently, the Claisen–Schmidt condensation appears to follow the broader trends observed across sustainable organic synthesis rather than representing an exceptional case, reinforcing the need for harmonized methodologies and transparent reporting when assessing the sustainability of alternative synthetic strategies.

Finally, it should be recognized that in organic synthesis, “green” methodologies often emerge from incremental improvements rather than radical transformations. Within this context, the rational design of more sustainable Claisen–Schmidt condensations will likely depend on the combined optimization of catalyst efficiency, solvent minimization, and energy input, supported by transparent and quantitative evaluation frameworks. Such an integrated and quantitatively grounded approach will be essential to move from nominally “green” protocols toward genuinely sustainable chemical processes and to promote evidence-based decision making in the design of future synthetic methodologies.

## 6 Conclusion

This mini review critically examined recent sustainable methodologies for chalcone synthesis via Claisen–Schmidt condensation through the application of quantitative green chemistry metrics. The comparative analysis revealed that solvent-free catalytic systems generally provide the most favorable balance between reaction mass efficiency, process mass intensity, and E-factor, whereas the use of solvents and high catalyst loadings frequently dominates the material footprint of the process. The results also demonstrate that improvements in isolated yield do not necessarily translate into enhanced overall sustainability, emphasizing the importance of evaluating multiple complementary metrics simultaneously. Furthermore, the analysis highlights significant limitations associated with inconsistent reporting practices, differences in system boundaries, and the limited availability of energy-consumption data. Collectively, these findings indicate that meaningful assessment of sustainable synthetic methodologies requires transparent reporting and standardized evaluation criteria. The integration of mass-based and energy-based metrics offers a valuable framework for identifying genuinely sustainable approaches and for guiding the future development of greener Claisen–Schmidt condensations and related carbon–carbon bond-forming reactions. Importantly, the complementary interpretation of reaction- and process-oriented metrics provides a more comprehensive assessment of sustainability than any individual metric alone.
