# Supplementary material for: Green metrics in chalcone synthesis via Claisen-Schmidt condensation: a critical mini review
Source: Front Chem. 2026 Sep 1;14:1909081. doi: 10.3389/fchem.2026.1909081 (PMC13576254; doi:10.3389/fchem.2026.1909081)
Supplement: Supplementary file 1 [file Supplementaryfile1.pdf]

Green metrics in chalcone synthesis via Claisen-Schmidt condensation: a critical mini review

Pamela Mendioroz[1], M. Julia Castro[2], Vanina Estrada[1,3], Darío C. Gerbino[2]*

[1] Planta Piloto de Ingeniería Química-PLAPIQUI (Universidad Nacional del Sur-CONICET), Camino La Carrindanga Km 7, 8000 Bahía Blanca, Argentina

[2] Instituto de Química del Sur, (INQUISUR, CONICET-UNS). Departamento de Química, Universidad Nacional del Sur. Avenida Alem 1253, 8000 Bahía Blanca, Argentina

[3] Departamento de Ingeniería Química, Universidad Nacional del Sur (UNS). Avenida Alem 1253, 8000 Bahía Blanca, Argentina.

* Corresponding Author: dgerbino@uns.edu.ar

Supplementary Information (S1) Calculation protocol for green chemistry metrics................ 2
S1. Scope of the calculations... 2
S2. Equations.. 2
Atom Economy ($\boldsymbol{AE}$).. 3
Reaction Mass Efficiency ($\boldsymbol{RME}$).. 3
Process Mass Intensity ($\boldsymbol{PMI}$) 3
E-factor ($\boldsymbol{E}$) 3
Specific Energy Consumption ($\boldsymbol{SEC}$) 3
S3. Treatment of recyclable catalysts... 3
S4. Set of metric calculations 3
S5. Assumptions and criteria... 4
References.. 4

Supplementary Information (S1)
Calculation protocol for green chemistry metrics

S1. Scope of the calculations

Among the metrics proposed in the *Green Chemistry Metrics Toolkit* (McElroy et al., 2015), this review focuses on AE, RME, PMI, and E-factor because these could be consistently recalculated from the experimental information reported across the selected studies. In addition, SEC was estimated to evaluate the energy demand of the different methodologies using a common calculation protocol. For comparative purposes, calculations were preferentially based on the benchmark Claisen-Schmidt condensation between benzaldehyde and acetophenone, as this reaction was the most consistently reported across the selected studies.

Although several publications described broader substrate scopes or higher-performing examples (e.g., biomass-derived aldehydes or substituted aromatic substrates), these were not used for comparative calculations unless the benchmark reaction was unavailable. This approach was adopted to minimize the influence of substrate-dependent effects and to enable a more meaningful comparison of the different catalytic systems.

The calculations were restricted to the synthesis stage and therefore excluded work-up, purification, and product isolation, irrespective of the original publication. Accordingly, only materials employed during the reaction itself (stoichiometric reactants, catalysts, solvents, and additives) were considered, whereas downstream operations (e.g., extraction, washing, recrystallization, chromatography, drying, and analytical procedures) were excluded from the calculations. Whenever experimental information was incomplete, the assumptions adopted are explicitly indicated.

S2. Equations

Notations:

- M_P stoichiometric products;
- M_R stoichiometric reactants;
- n: number of reaction cycles;
- $m_{R,i}$: total mass of stoichiometric reactants used in cycle i;
- $m_{S,i}$: total mass of solvents inputs used in cycle i;
- $m_{A,i}$: total mass of auxiliary inputs used in cycle i;
- $m_{P,i}$: total mass of product obtained in cycle i;
- m_C: catalyst mass;
- P: effective equipment power (kW);
- t: effective operating time (h);
- m_P: mass of product obtained (kg).

For recyclable catalysts, m_C was counted only once, provided that the same catalyst charge was reused experimentally without replacement. Reactants, solvents and additives were considered separately for each reaction cycle when appropriate.

Atom Economy (AE)

$$AE = \frac{\sum M_P}{\sum M_R} \times 100$$

Reaction Mass Efficiency (RME)

$$RME = \frac{\sum_{i=1}^{n} m_{P,i}}{\sum_{i=1}^{n} m_{R,i}} \times 100$$

Process Mass Intensity (PMI)

$$PMI = \frac{\sum_{i=1}^{n}(m_{R,i} + m_{S,i} + m_{A,i}) + m_C}{\sum_{i=1}^{n} m_{P,i}}$$

E-factor (E)

$$E = \frac{\sum_{i=1}^{n}(m_{R,i} + m_{S,i} + m_{A,i}) + m_C - \sum_{i=1}^{n} m_{P,i}}{\sum_{i=1}^{n} m_{P,i}}$$

Specific Energy Consumption (SEC)

$$SEC = \frac{P\ x\ t}{\mathrm{m}_P}$$

S3. Treatment of recyclable catalysts

For methodologies without catalyst recycling (n =1), the equations reduce to their conventional definitions. As previously mentioned, when catalyst reuse was experimentally demonstrated, catalyst mass was counted only once, whereas reactants, solvents, additives and product mass were accumulated over all reported reaction cycles. No assumptions regarding catalyst lifetime or recovery efficiency were made beyond the reported experimental evidence.

S4. Set of metric calculations

To facilitate transparency and reproducibility, the complete set of green chemistry metric calculations is provided as an Excel workbook (Supplementary File S2). The workbook follows the calculation protocol described in this document and is based on the metric definitions proposed in the Green Chemistry Metrics Toolkit by Clark et al. (McElroy et al., 2015), while incorporating the harmonized system boundaries and catalyst reuse criteria adopted in this review.

The workbook contains the complete calculations for all representative Claisen-Schmidt condensation methodologies evaluated in this review. For each methodology, the original experimental data were compiled from the corresponding publication.

S5. Assumptions and criteria

Solvent masses were calculated from the reported volumes whenever only volumetric data were provided, using solvent densities reported by the corresponding supplier or standard chemical reference (e.g., Fisher Scientific) when not explicitly specified in the original publication. The reaction scale was taken directly from the original publication. For SEC calculations, the effective operating time was taken from the reported reaction time. Equipment power corresponded to the experimentally reported value whenever available; otherwise, it was estimated from the equipment specifications or representative operating conditions, and the corresponding assumption is explicitly indicated. Catalyst recovery and catalyst reuse were considered only when explicitly demonstrated experimentally, and no assumptions regarding catalyst lifetime or recovery efficiency were made beyond the reported evidence.

References

McElroy, C. R., Constantinou, A., Jones, L. C., Summerton, L., & Clark, J. H. (2015). Towards a holistic approach to metrics for the 21st century pharmaceutical industry. Green Chemistry, 17(5), 3111-3121. https://doi.org/10.1039/c5gc00340g
